# Investigating the Average Glandular Dose and Exposure Parameters in Mammography Based on Compressed Breast Thickness and Imaging Projection: A Single-Centre Study in Jeddah, Saudi Arabia

**DOI:** 10.3390/healthcare14091248

**Published:** 2026-05-06

**Authors:** Dalal Alamoudi, Amna Babgi, Lama Bazuhayr, Shaza Alsharif, Amani Y. Alhalwani, Doaa Alamoudi

**Affiliations:** 1 College of Science and Health Professions, King Saud bin Abdulaziz University for Health Sciences, Jeddah 21423, Saudi Arabia; 2 King Abdullah International Medical Research Center, Jeddah 22384, Saudi Arabia; 3 College of Applied Medical Sciences, King Saud bin Abdulaziz University for Health Sciences, Jeddah 21423, Saudi Arabia; 4 Radiology Department, Ministry of the National Guard—Health Affairs, Jeddah 21423, Saudi Arabia; 5 Faculty of Management of Science, Yanbu Industrial College, Yanbu 46452, Saudi Arabia

**Keywords:** mammography, average glandular dose, compressed breast thickness, exposure parameters, Saudi Arabia

## Abstract

Introduction: The mammary gland is highly sensitive to ionising radiation, making the average glandular dose (AGD) the most appropriate metric for breast dosimetry. With increasing participation in breast screening programmes, cumulative radiation exposure remains a concern. This study aimed to investigate the correlation between AGD and exposure parameters under controlled conditions of compressed breast thicknesses and mammographic projections. Methods: A retrospective, cross-sectional analysis evaluated data from 609 patients who underwent clinically justified digital mammography examinations with a single direct digital radiography system at King Abdulaziz Medical City, Jeddah, Saudi Arabia, between September 2023 and September 2025. The Shapiro–Wilk normality test indicated that AGD data were not normally distributed. Consequently, the Wilcoxon signed-rank test was used to assess differences between craniocaudal (CC) and mediolateral oblique (MLO) projections for the right and left breasts across CBT ranges. Spearman’s rank correlation coefficient was used to evaluate associations between AGD and exposure parameters (compression force (CF), mAs, and kVp) under controlled conditions of CBT ranges and projections. Results: AGD increased with CBT, rising from 1.36–1.39 mGy at 30–39 mm to 2.25–3.05 mGy at 70–79 mm. MLO projections consistently showed higher AGD than CC projections, with greater differences from 50–59 mm. Statistically significant differences were observed in projections across CBT. Spearman’s analysis demonstrated a significant positive correlation between AGD and CBT (*p* < 0.0001), which was strongest in the right MLO (ρ = 0.5082). Within the 50–59 mm range, AGD strongly correlated with mAs and moderately with kVp, but not with CF. Conclusions: AGD increases significantly with CBT, particularly in MLO projections. Within the 50–59 mm CBT range, mAs showed the strongest influence on AGD, while kVp had a moderate effect and CF was non-significant. These findings support dose optimisation through exposure parameter control and alignment with DRLs while maintaining image quality.

## 1. Introduction

Mammography is widely recognised as a critical screening tool for the early detection of breast cancer. It decreases rates of extensive surgery, including mastectomy, and improves treatment options [1,2,3,4]. The effectiveness of mammography screening varies by patient age, screening interval, and study design [5]. However, evidence indicates that mammography screening can decrease breast cancer mortality rates by up to 30% [2,3,6], highlighting its significant role in breast cancer management and public health strategies. Mammography is used either to screen asymptomatic patients or to diagnose those with symptoms. The demand for mammographic services is rising; therefore, the safe and effective use of this diagnostic tool is crucial [7,8]. At the same time, it is essential to recognise that despite its benefits, mammography involves exposure to ionising radiation. While the associated risk of breast cancer is considered low, it is important to minimise radiation doses to as low as reasonably achievable (ALARA) while maintaining high image quality [2,8,9,10,11].

Breast cancer shows different patterns across populations. In Saudi Arabia, it tends to affect women at a younger age, with a median age at diagnosis of 50 years compared to the United States, where the median diagnosis age is 62 years [12]. In addition, Saudi women are more likely to be diagnosed at a later disease stage, with 57.3% presenting with distant metastatic cancer compared with 37% of women in the United States [12]. In response to these challenges, a breast cancer screening programme was introduced in Saudi Arabia in 2012. This programme offers biennial screening for women aged ≥40 years and annual screening for high-risk women aged ≥30 years. Its primary goal is to improve early detection and intervention, ultimately enhancing breast cancer outcomes and prognosis for Saudi women [13]. Routine breast cancer screening or diagnostic mammography includes two standard projections for each breast: craniocaudal (CC) and mediolateral oblique (MLO) projections [14]. During mammography, the glandular breast tissue, which is radiosensitive, is exposed to ionising radiation; thus, mammography raises the risk of radiation-induced cancer [15,16,17,18,19].

According to the National Health Service Breast Screening Programme, the risk of radiation-induced cancer from a single mammographic screening, which includes both the CC and MLO projections, is approximately 1 in 20,000 [10]. This highlights the need to monitor and optimise radiation doses to ensure patient safety during mammography. The average glandular dose (AGD) is the most appropriate dosimetric measure for estimating the risk of radiation-induced cancer [1,6]. It represents the effective absorbed dose in the glandular tissue of a uniformly compressed breast, excluding the skin [18,20,21,22,23]. Although the AGD cannot be measured directly, its measurement is recommended by major international organisations, including the International Commission on Radiological Protection (ICRP), the International Atomic Energy Agency (IAEA), and the European Commission [24,25,26,27,28]. The AGD can be calculated from all exposure parameters for a specific examination, along with factors such as compressed breast thickness (CBT) [28]. Furthermore, breast compression during mammography reduces its thickness, thereby decreasing the radiation dose received by the patient [29].

Diagnostic reference levels (DRLs) are essential standards for monitoring and adjusting radiation doses in mammography. The ICRP defines DRLs as ‘investigation levels applied to easily measurable quantities, such as absorbed dose in air or tissue-equivalent material at the surface of a simple phantom or a representative patient’ [30]. When the radiation dose associated with a diagnostic procedure exceeds the established DRL, corrective dose-reduction actions are required to improve safety. Many countries have established DRLs to guide radiation dose optimisation and to minimise variability in mammography procedures [13,30,31,32]. DRLs are typically determined by the 75th percentile of the median dose across multiple systems [13,32]. In countries with robust quality assurance (QA) programmes, the 95th percentile may be used as the national DRL (nDRL) due to the narrow distribution of AGDs [7,13,32]. Therefore, establishing nDRLs is important for optimising radiation doses in medical imaging [12,31].

The Saudi Food & Drug Authority (SFDA) has established nDRLs for digital mammography in Saudi Arabia. They set an nDRL for two-dimensional (2D) mammography with a compression thickness of 40–50 mm at 1.44 mGy (mean glandular dose) [31]. The Australian Radiation Protection and Nuclear Safety Agency recommends that healthcare institutions set local DRLs that align with nDRLs when possible [33]. A recent report from the European Commission advises updating local DRLs at least every 3 years and nDRLs every 5 years [25,34]. Despite these guidelines, significant variation in DRLs persists both within and between countries. In the United States, the regulatory radiation dose limit per mammographic projection is set at 1.6 mGy for digital mammography [30]. In addition, the latest IAEA Human Health Series Publication No. 17 indicates that acceptable AGDs range from 1 mGy to 6.5 mGy depending on breast thickness, while the achievable AGD typically ranges from 0.6 mGy to 5.1 mGy [27].

Previous studies have reported that AGD in mammography is primarily influenced by CBT, exposure parameters (mAs and kVp), and projection type, with higher doses generally observed in thicker breasts and MLO projections [6,35,36]. Multi-system and population-based investigations also report that AGD varies with patient age, breast characteristics, and mammography system design, particularly due to differences in automatic exposure control (AEC) strategies [6,37,38]. Additional evidence highlights variability between system-reported and calculated doses, reinforcing the need for standardised dosimetry approaches [39]. However, most existing studies rely on broad CBT ranges or pooled analyses, limiting detailed assessment of dose–parameter relationships within clinically specific thickness intervals.

Radiation dose exposure is a key concern in mammography screening due to the repeated imaging in screening programmes, making accurate dose evaluation essential for compliance with national DRLs established by the Saudi Food and Drug Authority (SFDA). However, to our knowledge, locally derived DRLs remain limited in many regions, particularly in the Western Region of Saudi Arabia. While previous studies have evaluated AGD in mammography, few have combined CBT stratification with projection-specific analysis under routine clinical conditions. The present study investigates the relationship between AGD and exposure parameters under controlled conditions of CBT ranges and mammographic projections. By focusing on a narrower CBT, this study provides a more controlled evaluation of dose determinants and generates locally relevant data to support dose optimisation, contributing to DRL development.

## 2. Materials and Methods

### 2.1. Study Design

This retrospective, cross-sectional study was conducted at King Abdulaziz Medical City-Jeddah (KAMC-J) and analysed mammography data obtained from a single digital mammography unit located in the Radiology Department.

### 2.2. Ethical Approval

Ethical approval was granted by the Institutional Review Board at King Abdullah International Medical Research Center, Jeddah, Saudi Arabia (NRJ24/001/11). This study did not require written informed consent for participation, according to national legislation and institutional requirements.

### 2.3. Mammography System Description

Mammography was performed using a Hologic Selenia Dimensions (Hologic, Inc., Marlborough, MA, USA) digital system equipped with a direct-conversion amorphous selenium (a-Se) detector with a pixel size of approximately 70 µm. The X-ray tube uses a tungsten (W) anode, with automatic selection of filter material for 2D imaging, typically rhodium or silver filtration when W is used. The automatic exposure control (AEC) mode was applied during all examinations to optimise exposure parameters, based on the breast thickness and tissue composition. The system underwent routine QA and control procedures in accordance with the manufacturer’s specifications and local regulatory requirements. The AGD was automatically calculated by the mammography system using the built-in Dance algorithm and displayed after each exposure [28]. The AGD values were retrieved from the Digital Imaging and Communication in Medicine (DICOM) header via the system software.

### 2.4. Patient Selection

Patient data were retrospectively collected from female patients aged 30–91 years who underwent standard four-projection mammography, including left CC (LCC), right CC (RCC), LMLO, and RMLO projections, between 6 September 2023 and 15 September 2025. The dataset included both screening and diagnostic mammography examinations. The mean patient age was 56 ± 10 years. A total of 1612 patients were initially identified, and data were extracted from the Digital Imaging and Communication in Medicine (DICOM) header during 2025. Exclusion criteria were applied to ensure consistency in imaging technique and reliability of comparisons across groups. These included the presence of breast implants, single-breast screening, use of combination images, non-standard number of projections (fewer or more than four), CBT exceeding 100 mm, limited sample sizes in the CBT categories, and compression force (CF) outside acceptable limits (i.e., 0 N or >200 N). Accordingly, 1005 patients were excluded, and 609 patients (2428 mammograms total) comprising four standard projections were included in the final analysis.

### 2.5. Data Collection

Exposure parameters and dose-related information were obtained from the DICOM files. Data on patient age, CBT (mm), kilovoltage peak (kVp), milliampere-seconds (mAs), force compression, target-filter combination, AEC mode used, AGD per projection (mGy), and entrance surface dose (ESD; mGy) were recorded in Microsoft Excel 2016 (64-bit; Microsoft Corporation, Redmond, WA, USA).

### 2.6. AGD and DRL Estimation

Average glandular dose (AGD), also referred to as mean glandular dose (MGD), represents the radiation dose absorbed by radiosensitive glandular breast tissue and is the recommended dosimetric quantity for risk assessment in mammography [40]. The AGD is estimated indirectly from the Entrance Surface Air Kerma (ESAK, mGy) and half-value layer (HVL).

AGD was estimated from ESAK and conversion coefficients derived from Monte Carlo simulations for standard breast projections, assuming a composition of 50% glandular and 50% adipose tissue. The breast consists of glandular, fatty, and fibrous tissues, and its composition varies with age. These coefficients depend on breast thickness, HVL, and exposure parameters, including tube voltage (kVp), target material, and filtration. The methodology for AGD estimation has been previously described by Sulieman et al. [40], using conversion factors developed by Dance et al. [28].

The dose was calculated according to equation:

AGD = ESAK × g × c × s

where

- g is the conversion factor from ESAK to glandular dose for a standard breast composition;
- c is a correction factor accounting for variations in breast composition;
- s is a correction factor for differences in the X-ray spectrum.

The g and c factors were selected based on HVL, beam filtration, and breast density, as reported in previous studies [28,41].

For the estimation of diagnostic reference levels (DRLs), and in accordance with international recommendations, the DRL was defined as the 75th percentile of the AGD distribution.

### 2.7. Statistical Analysis

Descriptive statistics were calculated, including the mean and standard deviation (SD), median, and interquartile range (IQR). A normality test was performed using the Shapiro–Wilk test on the AGD data collected from the right and left breasts, with different projections (CC, MLO) across different CBT ranges, which indicated that the data were not normally distributed. Consequently, the Wilcoxon signed-rank test was used to analyse numerical data and determine significant differences between the right CC and right MLO projections, as well as between the left CC and left MLO projections. Correlations between AGD and exposure parameters (CF, mAs, and kVp) at different CBTs were evaluated using Spearman’s rank correlation test for non-normally distributed data. All statistical analyses were performed using Python 3.12, and *p* < 0.05 indicated statistical significance.

## 3. Results

In total, 609 mammography examinations performed using the Hologic Lorad Selenia system were analysed (Table 1). The mean CBT was 55 ± 14.7 mm, the CF was 109 ± 9.76 N, the tube voltage was 30 ± 9.54 kVp, the tube current–time product was 149 ± 10.08 mAs, the ESD was 6.9 ± 3.98 mGy, and the AGD was 2.14 ± 0.50 mGy. The 75th percentile AGD was 2.24 mGy.

Table 2 compares the findings of the right (CC and MLO) and left (CC and MLO) breasts across CBTs ranging from 30–39 mm to 70–79 mm. The AGD increased progressively with CBT across all projections. For both right and left breasts, MLO projections showed higher AGD than CC projections within each CBT group. The differences between CC and MLO projections were statistically significant across all CBT ranges using the Wilcoxon signed-rank test, with *p*-values ranging from 0.0321 to <0.0001. Median values followed similar patterns to the means across all groups. The IQR increased with CBT, indicating greater variability in AGD at higher thickness ranges, particularly in MLO projections. Comparable trends were observed between right and left breasts.

Across most CBT categories, MLO projections (RMLO and LMLO) consistently demonstrated higher AGD values than CC projections (RCC and LCC), with the difference becoming more pronounced from the 50–59 mm range. At a CBT of 50–59 mm, the mean AGD was higher in the right breast for both CC and MLO projections (1.97 ± 0.41 mGy and 2.17 ± 0.33 mGy, respectively) compared with the left breast for both CC and MLO (1.85 ± 0.38 mGy and 2.14 ± 0.29 mGy, respectively). The differences between CC and MLO projections were highly significant (*p* < 0.0001).

For a CBT of 60–69 mm, the mean AGD values in the right breast were 2.17 ± 0.42 mGy (CC) and 2.67 ± 0.35 mGy (MLO), compared with 2.23 ± 0.33 mGy (CC) and 2.63 ± 0.37 mGy (MLO) in the left breast, with statistically significant differences between projections (*p* < 0.0001).

Similarly, at a CBT of 70–79 mm, AGD values were 2.25 ± 0.61 mGy for CC and 3.02 ± 0.61 mGy for MLO in the right breast, while the corresponding values in the left breast were 2.27 ± 0.68 mGy and 3.05 ± 0.69 mGy, with significant differences observed (*p* = 0.0321 and 0.0003, respectively).

**Figure 1:**
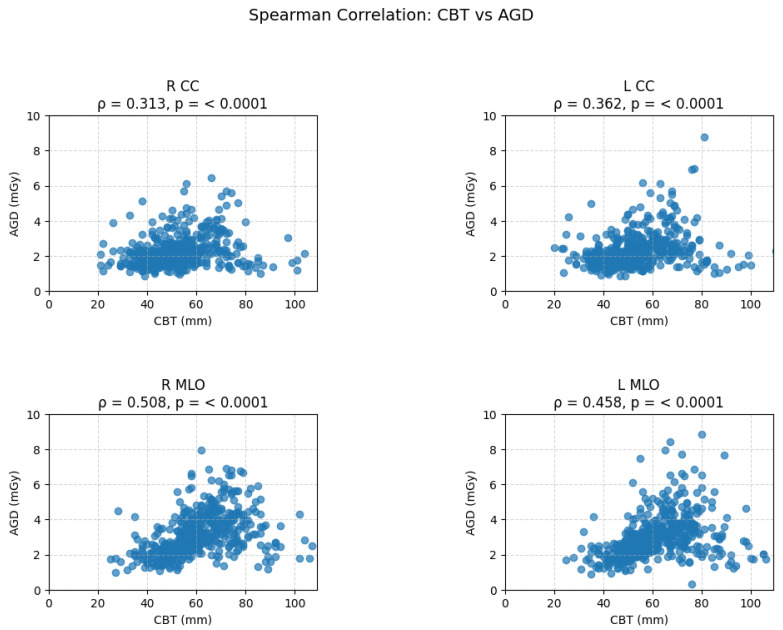
Spearman’s correlations between the average glandular dose (AGD) and compressed breast thickness (CBT, mm) with different projections (RCC, LCC, RMLO, LMLO).

Spearman’s correlation analysis demonstrated a statistically significant positive association between AGD and CBT across all mammographic projections (*p* < 0.0001), as shown in Figure 1. In the CC projections, a weak-to-moderate positive correlation was observed for the right CC (RCC) projection (rho = 0.3132) and a moderate positive correlation for the left CC (LCC) projection (rho = 0.3625), indicating that AGD increases with CBT. However, the relationship is relatively modest in these projections. Stronger correlations were found in the MLO projections, with the right MLO (RMLO) showing the highest correlation (rho = 0.5082) and the left MLO (LMLO) demonstrating a moderate-to-strong correlation (rho = 0.4581).

Overall, the results confirm a consistent and statistically significant positive monotonic relationship between CBT and AGD, with thicker breasts requiring higher radiation doses, particularly in MLO projections. Additionally, the strongest correlation was in the CBT range, 50–59 mm, RMLO projection (ρ = 0.358), and the weakest in the 50–59 mm LMLO projection (ρ = 0.200). With an increasing CBT range of 60–69 mm, correlations remain positive but slightly lower. Correlations are statistically significant (*p* < 0.05) in all cases.

Building on our previous finding that the 50–59 mm CBT range demonstrated the strongest overall correlation between CBT and AGD, further analysis was performed within the fixed CBT range to investigate which exposure parameters most strongly influence AGD in this projection. As shown in Table 3, Spearman’s rank correlation analysis revealed an extremely strong positive correlation between mAs and AGD in both MLO projections (ρ ≈ 0.96–0.995). A moderate positive correlation in RMLO and a weaker but significant one in LMLO were found between the AGD and kVp. In contrast, CF showed a weak, non-significant relationship with AGD in both projections (*p* > 0.05).

## 4. Discussion

The dosimetric and exposure parameters for the Hologic Lorad Selenia system evaluated in the current study provide a representative overview of routine clinical practice and patient dose exposure characteristics. The slightly higher AGD observed may be attributable to the AEC settings implemented in this system, given that Hologic units adjust exposure parameters based on breast density and compression. Previous research [13] has indicated that Hologic is the dominant mammography system vendor, with 99.6% of mammographs performed using AEC in Saudi Arabia. Specifically, the Hologic AEC requires approximately 2–10 mAs per exposure to determine the appropriate CBT and achieve the target detector dose [13].

Despite the observed AGD increase in the current study, the doses remain within established safety limits for mammography. The mean kVp and mAs reflect appropriate exposure settings selected by the AEC system to accommodate variations in breast thickness and composition, resulting in corresponding mean ESD and AGD values. Importantly, the AGD upper quartile (75th percentile) of 2.24 ± 0.50 mGy lies within internationally recommended DRLs for digital mammography [34]. The relatively small difference between the mean AGD and the 75th percentile suggests a narrow dose distribution, indicating stable system performance and consistent imaging technique. Overall, these findings demonstrate that the Hologic Lorad Selenia system operates within acceptable dose limits while maintaining appropriate exposure conditions. This supports the suitability of the measured AGD values for use in local DRL establishment and highlights the effectiveness of current dose optimisation practices.

CBT is one of the main determinants of the AGD. As expected, the AGD noticeably increased with increasing breast thickness in the present study. This reflects the higher exposure settings required to penetrate denser tissue and maintain good image quality. The concurrent AGD and CBT increases for both the MLO and CC projections align with the basic principles of X-ray imaging and with findings from previous studies [8,32]. Thicker breasts require higher exposure settings (mAs and kVp) to maintain image quality, resulting in greater radiation dose exposure. The higher AGD in MLO compared to CC projections, as noted in a previous study [8], is due to the larger tissue volume included, particularly glandular and pectoral muscle tissues, and the longer X-ray path through the breast in the oblique orientation [6,35]. This difference is more pronounced in thicker breasts, where tissue attenuation is greater. Despite the higher AGD in MLO projections, the values remained within acceptable DRLs, indicating that the imaging system maintains effective dose optimisation. Continuous monitoring of AGD across various breast thicknesses is essential to ensure compliance with radiation safety standards while maintaining image quality for accurate diagnosis. At higher CBT, the increasing differences in AGD lead to stronger statistical significance in *p*-values. When compared to the Saudi nDRL for digital mammography (AGD = 1.44 mGy in CC projection at 40–50 mm CTB, SFDA 2025) [31], the mean AGD identified in the present study in the CC projection at 40–49 mm CBT, (1.63 mGy ± 0.27) from RCC and (1.59 mGy ± 0.28) from LCC, was slightly higher. This indicates that the radiation doses administered in this study were acceptable, although slightly higher than the national reference value. The difference may reflect variations in breast composition, imaging protocols, or automatic exposure settings. Overall, the results align with the common trend in mammography practice, where the AGD increases proportionally with CBT to maintain image quality consistent across different tissue densities.

Across both projections evaluated in the present study, CBT demonstrated a significant positive association with the AGD, with the relationship being particularly strong in the MLO projection. These findings corroborate previous research [8,35,42,43] demonstrating that thicker breasts require higher dose exposure to achieve adequate image quality. Collectively, these results highlight the complexity of dose optimisation in mammography and suggest that managing breast thickness and adjusting exposure parameters may be more effective for dose reduction than increasing compression force alone. Therefore, optimisation strategies should prioritise achieving sufficient compression to ensure diagnostic image quality and patient comfort, rather than relying on compression force alone.

Based on the initial findings, the 50–59 mm CBT range was selected for further analysis, as it demonstrated the strongest association between CBT and AGD and included the highest number of patient observations within this range. Restricting the analysis to this narrower range reduced variability and allowed for a more controlled examination of the influence of exposure-related factors on radiation dose. The analysis also focused on MLO projections, where a consistent positive relationship between CBT and AGD was observed, with higher doses associated with increasing breast thickness. This approach allows for a focused investigation of the relationship between exposure parameters and dose under routine clinical conditions, while controlling for CBT range and projection.

### 4.1. Correlation Analysis Between the AGD and Exposure-Related Factors

Correlation analysis between AGD and exposure-related parameters was performed under controlled conditions of CBT range and projection. The strong correlation between the AGD and mAs across the CBT range reflects the dependence of dose estimation on exposure parameters used during mammographic image acquisition. This finding is consistent with those of previous studies [6,40,44,45] and aligns with established dosimetric principles, as mAs directly determines the number of X-ray photons contributing to the radiation dose delivered to the breast tissue. Although appropriate mAs selection is essential for achieving optimal image quality, particularly through improving the signal-to-noise ratio and enabling the visualisation of subtle anatomical details, excessive mAs can lead to unnecessary dose increases [6]. Therefore, careful optimisation of mAs is critical to balance image quality requirements with radiation dose reduction. Radiologic technologists should aim to use the lowest mAs that still maintains diagnostic image quality—a principle consistent with the ALARA standard.

The moderate correlation observed between the AGD and kVp in the current study supports previous findings [6]. However, while some studies have demonstrated a very strong positive correlation between the AGD and kVp [40], others have reported a very weak correlation [44]. These discrepancies may be attributed to differences in mammography systems, exposure protocols, AEC settings, and patient characteristics. The moderate role of kVp suggests that increasing tube voltage modestly might reduce the need for higher mAs, potentially balancing image penetration with acceptable dose, especially for thicker breasts. This trade-off can be optimised using automatic exposure control (AEC) systems or by adjusting technique charts based on patient size and tissue composition.

CF and CBT are closely linked in the context of mammography, with appropriate compression playing a critical role in reducing breast thickness, improving image quality, and minimising patient dose exposure [25]. Although adequate breast compression is known to reduce breast thickness and potentially lower dose exposure, the weak association between CF and the AGD identified in the present study indicates that CF alone may not significantly influence patient dose exposure without corresponding optimisation of exposure parameters. Our results demonstrate a weak, non-significant correlation between CF and the AGD within the 50–59 mm CBT range. This finding is consistent with previous findings [6,42,44,45] but differs from the findings of Waade et al. [43], who reported a positive correlation between CF and the AGD. Such discrepancies may be attributed to variations in breast characteristics, compression techniques, and imaging protocols across study populations [43]. As the analysis was limited to a narrow CBT range, the influence of CF on AGD appears minimal once adequate compression is achieved. This aligns with those of previous studies [6,46,47] reporting that further increases in compression force beyond a certain threshold do not significantly reduce breast thickness or radiation dose. In contrast, some studies have reported an inverse relationship between CF and CBT, suggesting that higher compression could reduce breast thickness and potentially lower the AGD [48,49]. Other investigations [50] have reported a positive association between CF and CBT, indicating that beyond a certain limit, additional compression may not effectively reduce breast thickness.

This suggests that its main importance remains improving tissue separation and image clarity rather than directly influencing AGD. Adequate but comfortable compression should still be applied to reduce breast thickness variation, which indirectly helps keep the radiation dose consistent. Overall, these findings emphasise that optimal compression practice should focus on effective reduction in breast thickness rather than achieving a predetermined CF. Importantly, all measured doses were within internationally recommended limits and the established Saudi nDRLs, confirming adherence to radiation safety standards within the national breast cancer screening programme.

### 4.2. Limitations and Future Research Directions

The study was conducted at a single centre using a single mammography system, which may limit the generalisability of the findings to other institutions or equipment types. Nevertheless, the study was performed in one of the largest medical cities in western Saudi Arabia, providing valuable evidence to support optimisation of local practice and the development of future local or national DRL recommendations.

A further limitation is that the potential impact of mathematical coupling between AGD and the exposure parameters may have influenced the interpretation of results or the generalizability of the conclusions. In addition, the reported correlations should be interpreted with caution, as they do not imply causal relationships, particularly due to the absence of multivariate adjustment to control for potential confounding factors in the present analysis.

Future research should involve multicentre, prospective studies representing all regions of Saudi Arabia to establish nDRLs, including stratification by CBT. Such studies should also evaluate protocol optimisation strategies across diverse patient populations and imaging systems while assessing the clinical outcomes associated with radiation dose. These efforts would improve understanding of how local protocols and patient characteristics influence compliance with DRLs and strengthen the evidence base for best practices and national policy development.

In addition, future work should combine AGD assessment with breast composition, patient body mass index (BMI), and radiographer-dependent positioning techniques to improve the accuracy of individualised dose estimation and optimisation strategies.

## 5. Conclusions

In this study, we analysed a dataset of mammographic dose measurements from a single hospital, providing a reliable reflection of the performance of breast screening and diagnostic systems at King Abdulaziz Medical City, Jeddah, Saudi Arabia. The findings show a consistent positive association between CBT and AGD, with thicker breasts requiring higher radiation doses, particularly in MLO projections. Within the selected CBT range (50–59 mm), AGD was primarily influenced by mAs, while kVp showed a moderate but significant association. In contrast, CF demonstrated a non-significant correlation with AGD, indicating a limited impact on dose once adequate compression is achieved. Overall, this study highlights the importance of ongoing quality assurance and dose optimisation in mammography, alongside regular comparison with national and international DRLs to ensure compliance with the ALARA principle while maintaining high diagnostic image quality.

## Figures and Tables

**Table 1:** Mammography examination parameters and radiation dose metrics obtained using the Hologic Lorad Selenia system.

Model	Number of Exams	CBT Mean (mm) (SD)	CF Mean (N) (SD)	kVp Mean (SD)	mAs Mean (SD)	ESD (mGy) (SD)	AGD (mGy) (SD)	75th (mGy)
Hologic Lorad Selenia	609	55 (±14.7)	109 (±9.76)	30 (±9.54)	149 (±10.08)	6.9 (±3.98)	2.14 (±0.5)	2.24 (±0.5)

**Table 2:** Inferential assessment of AGD per projection, right and left (CC/MLO) breasts at different CBTs ranges.

Projection	RCC			RMLO			*p*-Value *	LCC			LMLO			*p*-Value *
CBT	AGD			AGD				AGD			AGD			
	Mean (SD)	Median	IQR	Mean (SD)	Median	IQR		Mean (SD)	Median	IQR	Mean (SD)	Median	IQR	
30–39	1.36 (0.17)	1.4	0.85–1.48	1.39 (0.12)	1.36	1.28–1.4	0.0098	1.39 (0.16)	1.39	1.3–1.52	1.38 (0.18)	1.33	0.9–1.4	0.0215
40–49	1.63 (0.27)	1.61	1.44–1.86	1.68 (0.26)	1.66	1.5–1.9	0.0001	1.59 (0.28)	1.62	1.42–1.78	1.61 (0.25)	1.65	1.49–1.78	0.0044
50–59	1.97 (0.41)	1.96	1.68–2.32	2.17 (0.33)	2.23	2–2.4	<0.0001	1.85 (0.38)	1.88	1.56–2.13	2.14 (0.29)	2.15	1.94–2.33	<0.0001
60–69	2.17 (0.42)	2.23	1.70–2.50	2.67 (0.46)	2.8	2.49–2.94	<0.0001	2.23 (0.33)	2.27	2.01–2.49	2.63 (0.37)	2.69	2.37–2.99	<0.0001
70–79	2.25 (0.61)	2.01	1.63–2.70	3.02 (0.61)	3.49	2.78–4	0.0321	2.27 (0.68)	2.34	1.85–2.66	3.05 (0.69)	3.33	2.79–3.74	0.0003

* Wilcoxon signed-rank test, CBT in mm; AGD in mGy. Abbreviation: right breast (RCC/RMLO) and left breast (LCC/LMLO), interquartile range (IQR) [Q1–Q3].

**Table 3:** Spearman’s correlation between the average glandular dose (AGD) and exposure-related variables: tube current–time product (mAs), tube voltage (kVp), and compression force (CF) at a compressed breast thickness (CBT) range of 50–59 mm for the RMLO and LMLO projections.

Projection	Variable	mAs	kVp	CF
RMLO	Rho (ρ) coefficient	0.995	0.409	0.167
	*p*-value	<0.0001	<0.0001	0.0688
LMLO	Rho (ρ) coefficient	0.96	0.278	0.117
	*p*-value	<0.0001	0.0008	0.1868

## Data Availability

The original contributions presented in the study are included in the article materials. Further inquiries can be directed to the corresponding author due to privacy concerns.

## Abbreviations

The following abbreviations are used in this manuscript:

AGD	Average Glandular Dose
AEC	Automatic Exposure Control
ALARA	As Low As Reasonably Achievable
CBT	Compressed Breast Thickness
CC	Craniocaudal
CF	Compression Force
DRL	Diagnostic Reference Level
DICOM	Digital Imaging and Communication in Medicine
ESD	Entrance Surface Dose
ESAK	Entrance Surface Air Kerma
HVL	Half-Value Layer
kVp	Peak Kilovoltage
mAs	Milliampere-Second
MGD	Mean Glandular Dose
MLO	Mediolateral Oblique
NDRL	National Diagnostic Reference Level
PACS	Picture Archiving and Communication System
QA	Quality Assurance
